# Supplementary material for: Similarities and Differences between Psychosocial Determinants of Bullying and Cyberbullying Perpetration among Polish Adolescents
Source: Int J Environ Res Public Health. 2023 Jan 11;20(2):1358. doi: 10.3390/ijerph20021358 (PMC9859448; doi:10.3390/ijerph20021358)
Supplement: Supplementary file 1 [file ijerph-20-01358-s001.zip › ijerph-2082686-supplementary.pdf]

Table S1. Logistic regression predicting likelihood of bullying and cyberbullying perpetration in girls.

	Bullying						Cyberbullying					
	B	S.E.	P	Odds ratio	95% CI for Odds Ratio		B	S.E.	P	Odds ratio	95% CI for Odds Ratio	
					Lower	Upper					Lower	Upper
Grade (ref. older)	0.682	0.144	<0.001	1.979	1.491	2.626	0.770	0.166	<0.001	2.161	1.561	2.990
Family structure (ref. intact)	0.132	0.151	0.382	1.141	0.848	1.536	0.277	0.168	0.100	1.319	0.949	1.834
FAS (ref. low)			0.269						0.447			
FAS average	0.068	0.156	0.662	1.071	0.788	1.454	0.152	0.182	0.404	1.164	0.815	1.665
FAS high	-0.221	0.198	0.266	0.802	0.544	1.183	0.274	0.218	0.209	1.315	0.858	2.016
Deprivation quintile (ref. richest Q5)			0.598						0.355			
Deprivation index poorest (Q1)	0.141	0.220	0.521	1.152	0.748	1.772	0.114	0.258	0.658	1.121	0.676	1.859
Deprivation index (Q2)	0.334	0.211	0.113	1.397	0.924	2.113	0.140	0.255	0.581	1.151	0.699	1.895
Deprivation index (Q3)	0.147	0.195	0.453	1.158	0.790	1.698	0.430	0.217	0.047	1.538	1.006	2.351
Deprivation index (Q4)	0.048	0.194	0.805	1.049	0.717	1.535	0.271	0.215	0.208	1.311	0.860	1.997
School performance (ref. high)			0.052						0.010			
School performance low	0.506	0.208	0.015	1.658	1.103	2.493	0.709	0.247	0.004	2.033	1.254	3.296
School performance average	0.255	0.169	0.132	1.290	0.926	1.797	0.544	0.206	0.008	1.723	1.151	2.581
Social self-efficacy (ref. high)			0.013						0.636			
Social self-efficacy low	-0.609	0.213	0.004	0.544	0.359	0.826	-0.223	0.243	0.359	0.800	0.496	1.289
Social self-efficacy average	-0.396	0.171	0.021	0.673	0.481	0.941	-0.079	0.200	0.694	0.924	0.624	1.368
Empathy (ref. high)			<0.001						0.008			
Empathy low	0.965	0.223	<0.001	2.626	1.697	4.064	0.746	0.265	0.005	2.108	1.253	3.548
Empathy average	0.300	0.161	0.062	1.350	0.985	1.849	0.498	0.188	0.008	1.645	1.139	2.377
Life satisfaction (ref. high)			0.409						0.004			
Life satisfaction low	0.253	0.234	0.280	1.288	0.814	2.037	0.552	0.264	0.037	1.737	1.035	2.916
Life satisfaction average	0.051	0.202	0.800	1.053	0.708	1.565	-0.034	0.236	0.885	0.967	0.609	1.534
Family support (ref. high)			0.054						0.367			
Family support low	0.479	0.216	0.027	1.614	1.057	2.463	0.345	0.249	0.165	1.412	0.867	2.297
Family support average	0.177	0.196	0.366	1.193	0.813	1.751	0.179	0.224	0.425	1.196	0.771	1.856
Peer support (ref. high)			0.009						0.486			
Peer support low	0.290	0.208	0.164	1.336	0.889	2.009	-0.195	0.242	0.420	0.823	0.512	1.322
Peer support average	-0.223	0.170	0.190	0.800	0.573	1.117	-0.230	0.192	0.231	0.795	0.546	1.158
School attachment (ref. average or high)			0.749						<0.001			
School attachment very low	0.142	0.199	0.474	1.153	0.781	1.701	0.678	0.205	<0.001	1.970	1.318	2.944
School attachment rather low	-0.003	0.166	0.985	0.997	0.720	1.381	-0.155	0.199	0.435	0.856	0.580	1.264
Constant	-2.646	.329	<0.001	0.071			-3.841	0.399	<0.001			
R-sq. Nagelkerke			0.087						0.106			

Table S2. Logistic regression predicting likelihood of bullying and cyberbullying perpetration in boys.

	Bullying						Cyberbullying					
	B	S.E.	P	Odds ratio	95% CI for Odds ratio		B	S.E.	P	Odds ratio	95% CI for Odds ratio	
					Lower	Upper					Lower	Upper
Grade (ref. older)	0.521	0.124	<0.001	1.684	1.320	2.149	0.051	0.131	0.694	1.053	0.815	1.360
Family structure (ref. intact)	0.239	0.138	0.082	1.271	0.970	1.664	0.254	0.145	0.080	1.289	0.970	1.713
FAS (ref. low)			0.436						0.176			
FAS average	-0.134	0.142	0.346	0.875	0.662	1.155	0.223	0.155	0.151	1.250	0.922	1.695
FAS high	0.042	0.172	0.807	1.043	0.745	1.461	0.334	0.186	0.072	1.396	0.970	2.009
Deprivation index (ref. richest Q5)			0.022						0.857			
Deprivation index poorest (Q1)	0.418	0.185	0.024	1.519	1.056	2.185	0.082	0.195	0.673	1.086	0.741	1.590
Deprivation index (Q2)	0.480	0.195	0.014	1.616	1.102	2.369	0.012	0.205	0.954	1.012	0.677	1.512
Deprivation index (Q3)	0.327	0.181	0.071	1.387	0.973	1.979	-0.156	0.194	0.421	0.855	0.585	1.252
Deprivation index (Q4)	0.474	0.170	0.005	1.606	1.150	2.243	-0.084	0.184	0.647	0.919	0.640	1.319
School performance (ref. high)			0.597						0.651			
School performance low	0.137	0.181	0.450	1.147	0.804	1.635	0.075	0.191	0.693	1.078	0.742	1.566
School performance average	-0.020	0.147	0.889	0.980	0.735	1.306	-0.073	0.155	0.636	0.929	0.685	1.260
Social self-efficacy (ref. high)			0.996						0.592			
Social self-efficacy low	-0.005	0.195	0.980	0.995	0.678	1.460	0.150	0.210	0.474	1.162	0.770	1.754
Social self-efficacy average	-0.013	0.162	0.937	0.987	0.718	1.357	0.180	0.176	0.306	1.197	0.848	1.690
Empathy (ref. high)			0.060						0.031			
Empathy low	0.484	0.205	0.018	1.622	1.086	2.422	0.491	0.215	0.022	1.633	1.072	2.489
Empathy average	0.303	0.187	0.105	1.354	0.939	1.954	0.163	0.198	0.410	1.177	0.799	1.735
Life satisfaction (ref. high)			0.531						0.718			
Life satisfaction low	-0.203	0.212	0.338	0.816	0.538	1.237	0.125	0.221	0.571	1.134	0.735	1.748
Life satisfaction average	-0.155	0.152	0.309	0.857	0.636	1.154	-0.025	0.164	0.877	0.975	0.707	1.344
Family support (ref. high)			<0.001						<0.001			
Family support low	0.684	0.201	<0.001	1.982	1.337	2.938	0.822	0.211	<0.001	2.275	1.505	3.439
Family support average	0.550	0.164	<0.001	1.734	1.258	2.391	0.461	0.178	0.010	1.586	1.118	2.249
Peer support (ref. high)			0.507						0.976			
Peer support low	-0.051	0.204	0.802	0.950	0.636	1.418	-0.045	0.219	0.838	0.956	0.622	1.469
Peer support average	-0.170	0.170	0.316	0.843	0.605	1.177	-0.015	0.182	0.936	0.985	0.690	1.407
School attachment (ref. average or high)			0.001						0.015			
School attachment very low	0.627	0.198	0.002	1.873	1.271	2.760	0.547	0.201	0.007	1.729	1.165	2.566
School attachment rather low	0.373	0.156	0.017	1.452	1.070	1.971	-0.059	0.172	0.732	0.943	0.672	1.322
Constant	-2.207	0.296	<0.001	0.110			-2.314	0.317	<0.001	0.099		
R-Sq Nagelkerke	0.079						0.106					
